# Using a Heavy Inert Diffusion Additive for Superconformal Atomic Layer Deposition

**DOI:** 10.1021/acs.jpclett.4c03545

**Published:** 2025-02-26

**Authors:** Arun Haridas Choolakkal, Pamburayi Mpofu, Pentti Niiranen, Jens Birch, Henrik Pedersen

**Affiliations:** Department of Physics, Chemistry and Biology, Linköping University, SE-581 83 Linköping, Sweden

## Abstract

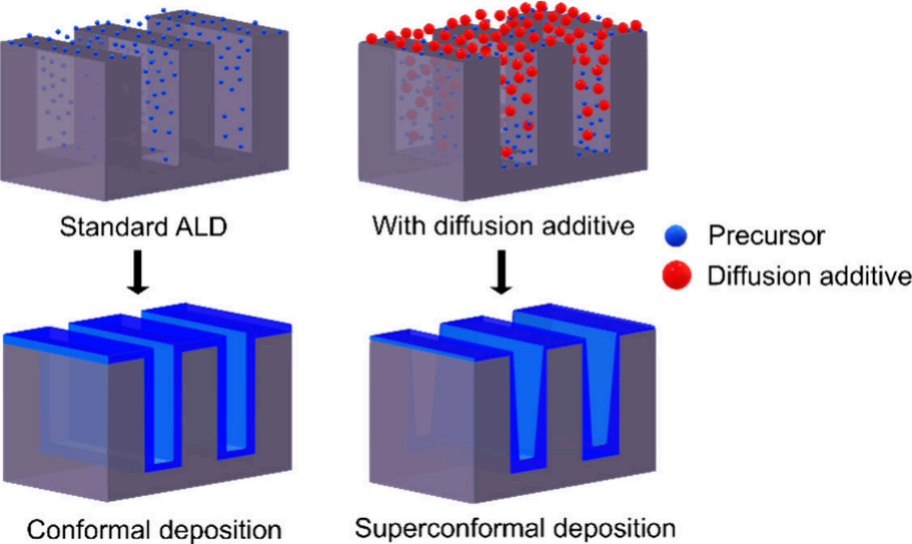

The shrinking of device nodes increases the demand for deposition processes to seamlessly fill nanometer-scale features. Despite the precision of atomic layer deposition (ALD), it cannot deposit in a V-shaped fashion, which is characteristic of superconformal thin-film deposition. We propose a strategy for superconformal ALD by adding a heavy inert gas as a diffusion additive. We show that the step coverage in an 18:1 aspect ratio feature increased from 1 to 1.6 with the addition of Kr in an ALD process for AlN from Al(CH_3_)_3_ and NH_3_. We speculate that the heavier Kr (84 amu) promotes diffusion of the lighter NH_3_ (17 amu) down the trenches. Consequently, NH_3_ molecules are pushed to the trench bottom, resulting in a lower growth per cycle at the trench openings. Further studies are needed to understand the effect of Kr, but we foresee that this approach to superconformal ALD is applicable to many ALD processes.

Deposition of thin, uniform layers, or films, of material onto topographically complex surfaces is essential to produce several semiconductor devices for logic and memory.^1^ The uniformity of a film in, *e.g*., a recessed feature such as a via is measured by the step coverage (SC) which is the ratio between the film thickness at the bottom of the feature and the film thickness at the top of the feature. A fully uniform film would render SC = 1, referred to as a conformal film. Atomic layer deposition (ALD) is generally regarded as the most competent technology for depositing conformal films^2^ and forms part of the backbone in modern semiconductor chip manufacturing.^3^ The key to the high conformality in an ideal ALD process is the use of sequential self-limiting surface chemical reactions with precursor molecules, typically supplied from the vapor phase. A sequential supply of, typically two, different precursors results in a process that alternates between adding one of two types of atoms to the surface in each reaction.^4^

However, if the film deposition requires filling of a recessed feature, such as a via or trench, a process that affords SC = 1 will not be sufficient. A perfectly conformal deposition will add a uniform layer of material in the feature to make it more and more narrow until the opening will be too narrow for molecules to diffuse down into the feature, and the film deposition pinches off the feature in the opening, leaving a hole in the middle of the feature. To properly fill a recessed feature, the deposition rate must instead be higher at the bottom of the feature than at the top. This will lead to an SC > 1, referred to as a superconformal deposition process.^5^

The most successful superconformal deposition processes have been chemical vapor deposition (CVD) processes. CVD relies, like ALD, on a supply of precursor molecules from the gas phase, but in CVD, the precursor supply is constant rather than sequential. This leads to a lower degree of control over the film deposition, as compared to ALD, and typically offers subconformal deposition, *i.e*., SC < 1. But CVD processes can be tuned to afford superconformal deposition.^5^ By carefully controlling the reaction rate and gas-phase collisions for the precursors, by using lower deposition temperature and lower total pressure, superconformal deposition zone diagrams have been developed.^6^ An alternative approach has been to add molecules that act as surface inhibitors to the CVD gas mixture. These molecules have a very high sticking probability to the surface and will therefore preferentially inhibit film deposition at the first surface they encounter, which is the opening of the recessed features.^7^

The sequential gas supply in ALD renders a perfectly conformal process but also limits the ability to afford SC > 1 as the supplied precursor vapor reaches all areas of the features and reacts to deposit a new layer of atoms. The concept of adding surface inhibitors has also been shown to be successful in ALD processes, affording filling of vias from the bottom up.^8^ Apart from that pioneering study, we are not aware of other attempts to make ALD produce films with SC > 1.

We recently presented an approach to increase the SC in CVD, without using inhibitors or low temperatures and pressures. Instead, we proposed to add an inert gas with a higher molecular mass than the precursor molecules to promote the diffusion of lighter precursor molecules down a trench. We demonstrated our concept with improved SC in a CVD process to deposit B_4_C from B(C_2_H_5_)_3_ by adding Xe.^9^ Here, we test the same approach in an ALD process, using deposition of AlN from Al(CH_3_)_3_ (72 amu) and NH_3_ (17 amu), as precursors, with a coflow of Kr (84 amu). We demonstrate how the addition of Kr can afford an ALD process with SC = 1.6 in a trench structure but that the Kr addition does not lead to longer deposition depth in a lateral high aspect ratio structure.

A hot-wall Picosun R-200 Advanced ALD reactor was used for the depositions. The reactor operated at a pressure of 4 mbar with a continuous flow of 400 sccm of high-purity N_2_ (99.999%, further dried using a getter filter), which served as purge gas. The reactor walls were heated to 350 °C and the substrate table was heated to 350 °C for all depositions. Trimethylaluminum (TMA), aluminum precursor (epivalence, electronic grade), was kept at 22 °C, and vapor was drawn by opening a valve to the reactor, which was at a lower pressure. The processes used NH_3_ (AGA/Linde, 99.999%, further purified by a getter filter) as the nitrogen precursor. From our previous study on this ALD process, in the same reactor,^10^ we used 0.1 s TMA pulse and 12 s NH_3_ pulse. The purge time after both TMA and NH_3_ pulses was 10 s. Depositions were conducted both without and with a diffusion additive. In the latter case, Kr (99.999%) with a flow of 15 sccm was used as diffusion additive during the whole process, *i.e*., not pulsed in with the precursors. Prior to deposition, Si(100) substrates that were 55 μm deep and 3 μm wide, *i.e*., 18:1 aspect ratio, trench structures, or lateral high aspect ratio (LHAR) Pillar Hall chips from Chipmetrics were used as substrates. The LHAR chips have an 0.5 μm opening to a 150 or 75 μm deep hall, enabling studies of up to 300:1 or 150:1 aspect ratio structures. The Si trench structures were cleaned with acetone and isopropanol for 3 min each and subsequently blow-dried with N_2_ gas; the LHAR chips were used as received.

The film thickness was measured by using scanning electron microscopy (SEM). Measurements were performed using a Zeiss Sigma 300 model. For the cross-section measurements, an in-lens secondary electron detector with 3 keV accelerating voltage and 30 μm aperture was used. SE2 secondary electron detector with the same parameters was used to study the top view of the LHAR chips. X-ray photoelectron spectroscopy (XPS) was employed to study the chemical environment of the film. The measurements were conducted using a Kratos AXIS Ultra DLD, equipped with an Ar^+^ sputter source. Monochromatic Al Kα X-ray radiation with a wavelength of 8.35 Å and a power of 150 W (anode current = 10 mA and anode voltage = 15 kV) was used to record the spectra. The films were subjected to sputter cleaning for 120 s at 500 eV Ar^+^ ion energy prior to the measurements. CasaXPS software was used to analyze the spectral data.

The cross-sectional SEM micrographs in Figure 1 illustrate the deposition of AlN thin films in the 18:1 aspect ratio trench structure without any addition of Kr. The ALD process rendered excellent conformality, *i.e*., SC = 1, with a film thickness of 39 nm at both the top and bottom of the trench structure. The observed film thickness was achieved after 500 ALD cycles, indicating a growth per cycle (GPC) of approximately 0.8 Å, in line with our previous study on this ALD process in the same ALD reactor.^10^

**Figure 1:**
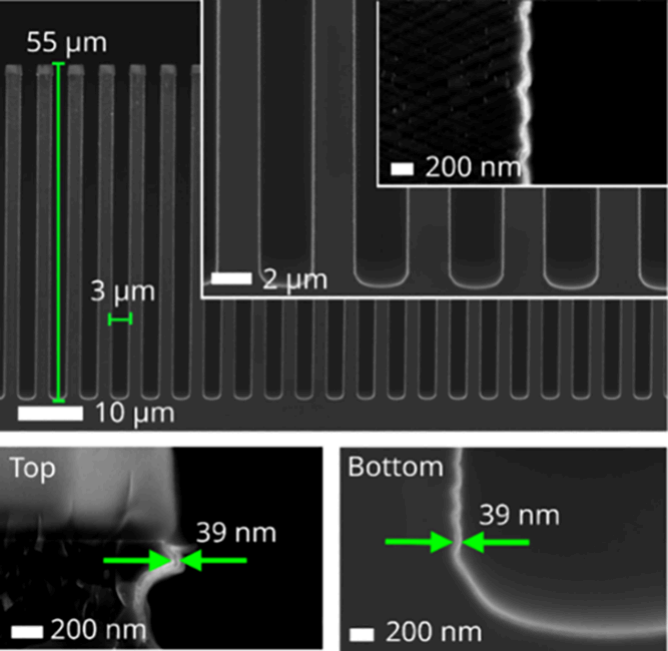
Cross-section SEM micrographs of AlN deposited without Kr added in trenches with an 18:1 aspect ratio. Conformal deposition with an SC of 1 was achieved after 500 ALD cycles, resulting in a uniform film thickness of 39 nm at both top and bottom of the trench structures.

The SEM micrographs in Figure 2 illustrate the step coverage of an AlN thin film deposited in the same type of trench structure but now with a coflow of Kr during the process. A film thickness of 24 nm at the top surface was achieved after 500 ALD cycles with a coflow of Kr, increasing to 39 nm at the bottom of the trench. This corresponds to SC = 1.6. As noted, the addition of Kr reduced the GPC at the top surface of the trench while maintaining the GPC at the bottom of the trench. This is similar to our previous result with Xe addition to a CVD process for B_4_C where the growth rate also decreased at the top of the trenches upon addition of Xe.^9^ It can be noted that in that study, we observed a somewhat higher growth rate at the bottom of the trenches.

**Figure 2:**
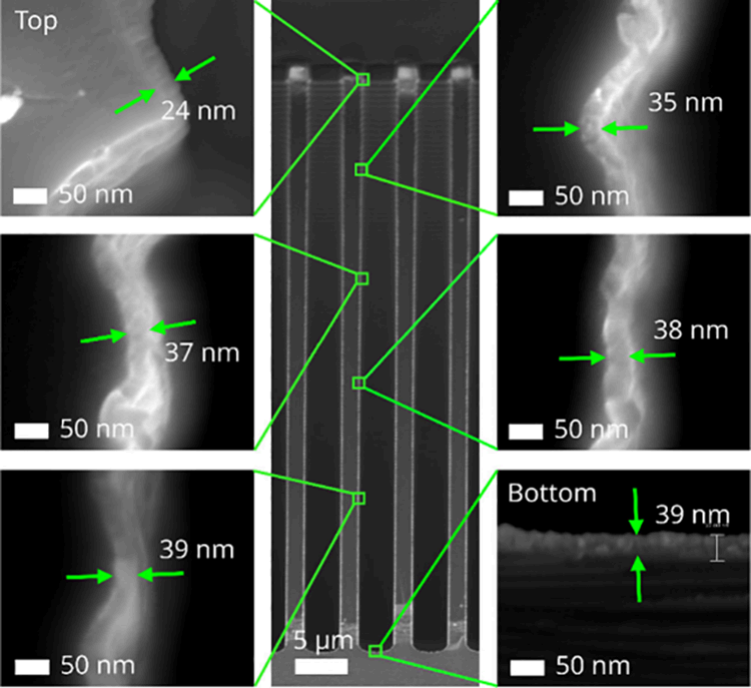
Cross-section SEM micrographs of AlN thin film deposited with a coflow of Kr in trenches with 18:1 aspect ratio. The process rendered a film thickness of 24 nm at the top surface and 39 nm at the bottom of the trench after 500 ALD cycles, corresponding to SC = 1.6.

We suggest that the difference noted between Figure 1 and Figure 2 can be discussed in terms of precursor diffusion.^9^ As the precursors are introduced into the reactor, their partial pressures instantly increase. Along the rising edge of the precursor partial pressure, the precursor molecules diffuse toward the bottom of the trench structure, with a driving force to even out local differences in partial pressures. From Graham’s law of diffusion, the diffusion velocities of two gases in thermal equilibrium depend on their molecular masses.^11^ Lighter molecules diffuse more quickly than heavier molecules. The small difference between the molecular mass of TMA (72 amu) and Kr (84 amu) should have a very small effect on the diffusion of TMA. But the large difference in mass between NH_3_ (17 amu) and Kr should, according to Graham’s law of diffusion, cause a significant difference in their diffusion down the trench. This would explain the reduction in GPC at the trench opening, as the nitridation of the TMA monolayer is reduced by the higher diffusion velocity of NH_3_, decreasing the partial pressure of NH_3_ at the trench opening. On the other hand, toward the bottom, the partial pressure of NH_3_ is maintained, or possibly increased, maintaining the GPC.

An alternative explanation is that as an inert Kr atom collides with the surface as it diffuses down the trench, the heavy Kr atom can act to enhance the surface desorption of adsorbed precursors. Especially in the case of lesser-reactive NH_3_, which takes more time to saturate the surface reaction. This would lead to a decreased GPC, and the desorbed precursors would be available for film deposition when they reach a new surface site. It could possibly be argued that this enhanced surface desorption by the Kr addition would render a lower GPC at the top of the trench while maintaining a saturated GPC (0.8 Å) closer to the bottom surfaces. We propose that the explanation for the observed results is likely a combination of, at least, these two factors and that further studies and modeling are needed to fully understand the results. The lower GPC at the opening of the trench is most likely not explained by a surface inhibiting effect from Kr, as the Kr atoms are expected to be inert.

The effect of the Kr coflow was further studied using lateral high aspect ratio (LHAR) structures with a removable top membrane. Top-view SEM was used to analyze the AlN deposition in the LHAR structures after removal of the top membrane (Figure 3). The analysis shows that both with and without the Kr coflow, the AlN deposition depth reached 42 μm into the structure in both 150 and 75 μm deep structures. This is equivalent to an aspect ratio of 84:1. We interpret these results such that the deposition depth is primarily limited by the TMA diffusion. We suggested above that the addition of Kr, *i.e*., the given time of the TMA pulse, in this case 0.1 s, sets the limit for the penetration depth.

**Figure 3:**
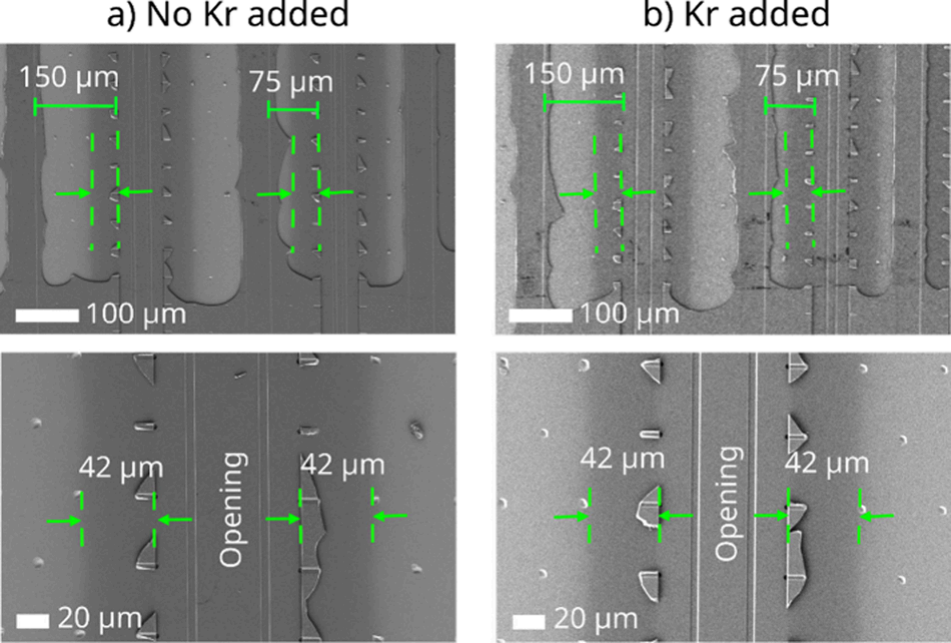
Top-view SEM micrograph of LHAR structures with the top membrane removed, following AlN deposition by (a) no Kr-added and (b) Kr-added processes. The top row displays lower-magnification images, illustrating deposition depth in 150 and 75 μm deep structures. The bottom row presents higher-magnification images.

Further, we investigated the chemical environment of the deposited film, both at the opening and within the structure after removal of the top membrane, as presented in Figure 4. The Al 2p XPS core-level spectra at the opening revealed two distinct peaks. The major peak, centered at 74.1 eV,^10^ was assigned to the Al–N bond, while the minor peak, centered at 72.5 eV,^10^ was assigned to elemental Al in the film. The small contribution of elemental Al is assumed to be sputter-induced reduction since NH_3_ cannot act as a reducing agent. The spectra obtained by probing within the structure also resulted in two distinct peak positions; the Al–N signal was slightly shifted to 74.2 eV, and the intensity of the elemental Al signal was reduced and shifted to 72.3 eV. The fwhm for the Al–N signal increased from 1.67 eV at the opening to 1.76 eV inside the structure, indicating more charging due to possible increase in the dielectric constant of the AlN.

**Figure 4:**
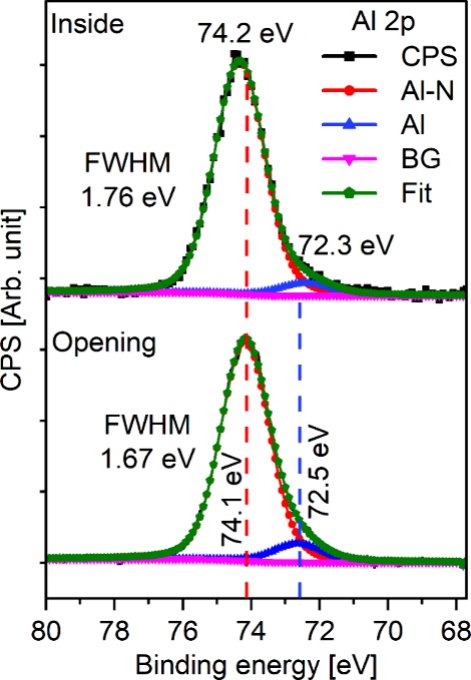
Al 2p XPS core-level spectra obtained at the opening and within the LHAR structure.

Finally, we see no reason why this approach to enhance the SC of an ALD process cannot be applicable to other ALD processes of similar kind. From Graham’s law of diffusion, we propose that the added gas must be heavier than the precursors that should diffuse further down the recessed features. The added gas must also be inert to the deposition chemistry to not leave any impurities in the film. Thus, the molecular weight and the chemistry of the precursors dictate which gases can be used as diffusion additives. In our previous study, we noted that the flow rate of the diffusion additive has an effect on the enhancement of the SC in CVD.^9^ Similar effects could possibly be found in ALD. We hope that future studies can uncover more details of the exact physical and chemical nature of this type of ALD process.

In conclusion, we show that it is possible to afford a superconformal ALD with a SC of 1.6 in 18:1 aspect ratio trench structure by adding a coflow of inert Kr gas to the ALD process. The SC increase is an effect of a lower GPC at the trench openings. We do not see a longer deposition depth in the LHAR structures with Kr addition. We suggest that the added Kr acts to promote the diffusion of the NH_3_ into the trenches and to enhance surface desorption, primarily at the trench openings. More studies are needed to fully understand the effect of the added inert heavy gas. We foresee that this approach to superconformal ALD can be applied to many ALD processes.
